# Perceived Physical Activity Levels and Objective Measures: A Mixed-Methods Study of Residents Aged 65 and Above in Assisted Living Homes in Australia

**DOI:** 10.1155/2024/9688105

**Published:** 2024-11-21

**Authors:** Samaher Alowaydhah, Ishanka Weerasekara, Sarah Walmsley, Sally Keir, Jodie Marquez

**Affiliations:** ^1^College of Health, Medicine and Wellbeing, The University of Newcastle, Newcastle, Australia; ^2^College of Applied Medical Science, Jouf University, Sakakah, Saudi Arabia; ^3^Faculty of Health and Social Sciences, Western Norway University of Applied Sciences, Bergen 5063, Norway; ^4^School of Allied Health Science and Practice, Faculty of Health and Medical Sciences, The University of Adelaide, Adelaide 5005, South Australia, Australia; ^5^Hunter Medical Research Institute, New Lambton, Australia

**Keywords:** accelerometer, assisted living home, exercise, geriatrics, physical activity

## Abstract

**Background and Purpose:** Communities face a mounting social, economic and health burden as the global population of older adults continues to grow. Regular physical activity is consistently reported as an effective means of maintaining health and independence in older adults, yet engagement in activity remains low. This study assesses the activity levels of adults aged over 65 years residing in Australian assisted living homes, and extended to examine their perception of their activity and explore possible factors that hinder or promote their engagement in physical activity.

**Methods:** Thirty-five older adults were recruited to this mixed-methods study from four separate assisted living homes. Objective activity data from five consecutive days of accelerometer wear were collected in combination with data from standardised and demographic questionnaires, and semistructured interviews. Qualitative data from interview transcripts were thematically analysed using NVivo software to develop themes relating to physical activity levels of older adults. Quantitative data from accelerometers and questionnaires were descriptively analysed, and associations between variables were examined using STATA software.

**Results and Discussion:** Findings indicated that females were more active than males, and those who were younger and those with lower body mass index (BMI) displayed higher activity levels. Additionally, residents in assisted living facility (number 4) and those who did not need ambulatory assistance are more active. Conversely, those utilising support services and those with more comorbidities demonstrated lower activity levels. Marital status and cognitive function did not show any association with activity levels of older adults. During the interviews, many older adults acknowledged the importance of physical activity, and some believed they were active. However, objective results contradicted this perception.

**Conclusion:** This study provides valuable insight into the demographic and health factors contributing to higher levels of activity, and the perceptions of activity among older adults vulnerable to health conditions associated with inactivity.

## 1. Introduction

In 2021, there were 761 million people aged 65 and above globally, and this number has been estimated to reach 1.6 billion by 2050 [1]. Between 1999 and 2019, the number of Australian people aged 65 and over increased from 12.3% to 15.9% [2], and this figure is also projected to continue to increase. Promoting and maintaining good health in this population is essential to limit the burden on both the individual and the health system.

There were 185,000 Australians using residential aged care services in 2022, and almost 800,000 older adults used home support programs during 2021–22 [3]. Many older adults live in assisted living homes, which offer transitional support for residents who do not need 24-h nursing care but still need some level of assistance, to maintain living independently [4]. Facilities in this category may be referred to as supported living homes, retirement homes and continuing care retirement communities. In general, residents uphold their autonomy, and most communities furnish amenities, activities and services [5]. These facilities may offer assistance in daily activities such as cleaning services, personal care, meal preparation and social activities, depending on the individual needs of the residents. These facilities are not regarded as medical institutions but must comply with state health and safety standards [6]. These communities provide a distinctive combination of camaraderie, independence, confidentiality and safety within a familiar environment [6]. In Australia, ‘assisted living home' is the term used to describe this level of support and will be referred to as such in this study.

Physical activity (PA) is at the centre of health promotion with a number of proposed health benefits for older adults, including improved balance, strength, functional independence, psychological state, bone density, gait tolerance, quality of life and reduced rate of falls [7–10]. The most recent PA guideline for older adults from the World Health Organization recommends 150 min of moderate intensity and 75 min of vigorous intensity PA per week [11, 12]. Despite strong recommendations for PA, it has been reported that over 30% of the older population do not engage in exercise [11]. Reported barriers to PA include fear of falling [13], fatigue [14], health problems such as chest pain and shortness of breath [15], diabetes, hypertension, musculoskeletal pain [16], inadequate time or encouragement and environmental barriers such as weather [16, 17].

Previous studies indicated that barriers to exercise included lack of opportunity to exercise, apathy and loss of independence, lack of awareness of the benefit of PA [18] and lack of motivation by health professionals who provide care to facilitate residents' PA [19]. Consequently, it is widely acknowledged that residents are sedentary the majority of their time [20]. This existing research provides foundational understanding of factors influencing PA in residential care in an Australian context; however, it is not known if this can be generalised in an Australian context or to represent residents of assisted living homes where there is an assumption of reduced activity that renders them a population at risk of inactivity-related health complications. A comprehensive understanding of current levels of activity, barriers and facilitators and perceptions of exercise will help develop safeguards for older adults at risk of transitioning to higher levels of residential care, with subsequent benefits to government financial resources. This study explores factors that influence the activity levels of older adults in assisted living homes in Australia, where individuals are seen as independent, yet susceptible to decline, and aims to inform the development of effective strategies to encourage PA among this population.

## 2. Methods

The underlying theory for this study is based on the socioecological model. This is a conceptual tool used to analyse the complex, interdependent interactions between social and ecological systems. The model proposes that individual behaviour, such as physical activity, is influenced by multiple levels of personal and environmental factors. This study design guides the exploration of how adults aged over 65 in Australian assisted living homes perceive their activity levels and identifies the personal, social and environmental factors that either hinder or promote their engagement in physical activity.

### 2.1. Research Aims and Questions

This study seeks to answer the following questions:

1. How physically active are residents in assisted living homes?
2. What are residents' perceptions of their own PA levels?
3. How do residents' perceptions of PA levels compare to their objective measures of activity?
4. What barriers and facilitators do residents report in engaging in PA?
5. What health and demographic variables are associated with PA levels among residents in assisted living homes?

### 2.2. Design

This study used a prospective observational design with a concurrent triangulation approach [21] to enable a comprehensive understanding of activity in Australian assisted living homes. We conducted concurrent triangulation by collecting quantitative data using accelerometers (StepWatch 4RE) to assess physical activity levels along with qualitative data through interviews to explore residents' perceptions of activity. The data from each method were analysed separately. Subsequently, the results from both analyses were compared to identify areas of agreement, discrepancies, or insights before interpretation. This approach allowed us to explore different facets of the research problem simultaneously, providing a fuller picture.

Discrepancies between quantitative and qualitative data provide deeper insights into need for further investigation.

Local assisted living homes, in the Greater Newcastle Region of New South Wales (NSW), Australia, were identified via online searches, and consent was sought from them for assistance in recruiting residents from their facilities for participation. Individual residents were screened for cognitive capacity to provide informed consent and recruited over a 12-month period. Ethical approval for this study was obtained from the University of Newcastle Human Research Ethics Committee (H-2021-0142) prior to commencement.

### 2.3. Setting

This study was conducted as a convenient selection in four assisted living homes in the Greater Newcastle Region NSW, Australia. Facilities were considered eligible if they met the definition of assisted living home: providing assistance to older adults requiring support with daily tasks and certain healthcare needs, but not offering 24 h care services as nursing homes do. Facilities were sequentially recruited until the target sample of 35 residents had been reached.

### 2.4. Sample Size Calculation

A power calculation was conducted to estimate the mean PA level of older adults living in care facilities (using a population standard deviation of 8.4 min/per day, 95% confidence within 3 min difference of the mean [22]), which determined that a minimum sample size of 31 residents was required for the study. Thirty-five residents were recruited to allow for lost data.

### 2.5. Residents

Residents were considered for inclusion in the study if they met the following criteria:

1. Aged 65 years or more.
2. Residing in an assisted living home, having been a resident for at least 3 months prior to enrolment.
3. Having capacity to provide informed consent as determined by a Mini-Mental State Examination Score (MMSE) greater than 17.
4. Not having an acute health condition that limited their current level of activity (e.g., recent fracture, surgery, delusion, etc.).
5. Comprehensive understanding of the English language.

### 2.6. Instruments

#### 2.6.1. Demographic and Baseline Information

Demographic information included gender, age, height, weight, education, assistive device use and marital status. Data regarding comorbidities were collected using the Charlson comorbidity index (CCI) [23, 24], and quality of life using the Older People Quality of Life Scale (OPQOL-35) [25] was also collected. The CCI classifies comorbid conditions which may influence mortality risk. This involves weighted scoring of the presence of 18 wide-ranging medical conditions including renal disease, cardiac disease, diabetes and cancer. The OPQOL-35 consists of 35 items to assess life overall, health, social relationships and participation, independence, control over life and freedom, home and neighbourhood, psychological and emotional well-being, financial circumstances, and culture and religion. A high score indicates a good quality of life [25].

Cognition was measured using the MMSE, where a score closer to 30 is indicative of higher cognitive function [26]. Any resident who scored < 17 was deemed ineligible for the study, due to cognitive impairment which may have affected their compliance with the research requirements and capacity to provide consent [27].

#### 2.6.2. Physical Activity Level

Habitual PA was objectively measured by accelerometry (StepWatch Activity Monitor [SAM]). This device has been frequently used in PA research with older adults and has been shown to be valid and reliable [28]. The test–retest reliability of accelerometry has previously been found to be excellent (ICC = 0.97) for older adults [29]. Residents were instructed to attach the accelerometer to their right ankle, via a strap, for 12 h during the day for five consecutive days [30], except during water activities. Previous studies have determined that 3–4 days of monitoring is sufficient to assess habitual PA; therefore, to allow for lost data and the inclusion of a weekend day, 5 days of monitoring was chosen [31]. Data were downloaded using ActiGraph software into a secured computer database for analysis.

Hourly mean PA counts per minute were studied for a 12-h period corresponding to hours of the day when residents were most likely to be out of bed [32]. PA variables recorded included: time (minutes per day) of total PA, percentage of time spent in different intensities of exercise (light, moderate and vigorous), number of steps per day, cadence and percentage of time inactive. The time spent at different levels of PA was determined using the following criteria: sedentary, with no walking; light, 1–15 steps per min; moderate, 16–40 steps per min; vigorous, 41 or greater steps per min.

#### 2.6.3. Activity Perception of Residents

Semistructured interviews were undertaken to ascertain the perceptions of residents regarding their level of activity and associated factors. An interview schedule was developed by the research team, piloted with a small sample and revised as required. The interview contained a combination of closed and open-ended questions, consisting of some questions about the older adult's level of activities: do they want to be more active; what types of activities they usually undertake; how much activity they do per week; what types of exercise they like to do and what obstacles decrease their level of activity? Other questions related to services that are provided for them by their facility that prevent or encourage them to be active.

All interviews were audio recorded and transcribed using Otter software [33]. Residents were offered the right to review their transcripts to verify accuracy prior to analysis.

### 2.7. Data Analysis

#### 2.7.1. Quantitative Analysis

We aimed to determine the impact of various factors, such as gender, comorbidity, marital status, facility, ambulatory assistance, services, age, education, body mass index (BMI), MMSE and QoL, on physical activity using the variables obtained from the step watch data. We used paired *t*-test to describe the data and multiple regression to investigate associations using physical activity measures as the dependent variable.

#### 2.7.2. Qualitative Analysis

Interviews were conducted with all 35 recruited residents and transcribed by the primary author. Interview data were analysed using the interpretative thematic analysis method of Braun and Clarke [34] and facilitated by NVivo software [35]. Both deductive (directly answering research questions) and inductive (exploring concepts in what was said) approaches were included in the development of codes and themes. Coding was conducted on 27 transcripts until it was deemed that saturation had been reached with no new codes being added. This process was undertaken with review and discussion of authors 3 and 4 with the primary author, until consensus was reached for the final codes and themes.

#### 2.7.3. Mixed Methods

A comparison of the activity levels recorded and residents' perceptions of activity levels was undertaken. A count of participants expressing each of the coded items was undertaken to reflect the frequency of various responses.

## 3. Result

### 3.1. Resident and Facility Characteristics

Thirty-five individuals residing in four separate assisted living home facilities participated. Their average age was 83.8 years, SD ± 7.20 (range 71–99), with 24 (68.5%) females and 11 (31.4%) males in our sample. Educational levels ranged from 8 residents not completing school to 10 attaining university degrees. To support independence, home cleaning was the most frequently sought assistance (57%), but 37% did not require any services. Most did not require any mobility aids (71.4%), and some had no disease (11%) or had a CCI score of mild (46%). Nineteen (54.2%) residents lived alone (see Table 1). Thirteen participants indicated an interest and were subsequently sent the full typed transcript of their interview. No comments or edits were returned to the researchers indicating acceptance of the accuracy and content of the transcripts.

There were notable differences between the facilities including geographical location, residential design and age of residents. Three of the four facilities consisted of single-level villas with courtyards located some distance from the city centre. The other facility was a vertical construction containing balconied apartments, and situated in the city centre, close to beaches, medical centres, restaurants, cafes, a gym and shopping centres. The residents in this facility were also significantly younger than those in the other three facilities with an average age of 79 ± 5.1 compared to 88 ± 6.3 years (*p*=0.002).

### 3.2. Measured Physical Activity

On average, our residents took 3940.31 steps daily, with a wide range from 1685.25 to 8316.4 steps. They dedicated 12% of their day to low activity, 6% to moderate activity and 1% was spent in vigorous activity. For 81% of the time, no PA was undertaken. The average cadence during the most intense continuous 60-min period of the day was 18.30 steps per minute, with an overall cadence average of 14.06 (see Table 2).

Females spent a larger portion of their day in low activity (Mean ± SD, 12.7 ± 2.8) compared to males (9.8 ± 3.6), with a significant between group difference (*p*=0.01). There were no further significant differences in measures of activity such as medium and high activities, steps per day or minutes active between the genders.

There was not any significant difference between residents who have no morbidities or one morbidity or more for all measurements of activity. Similarly, living alone or with a partner did not have any effect on physical activity level across any of the measured activity domains.

Residents living in the apartment style facility demonstrated higher levels of activity across a range of measures including minutes active (301 ± 84.7, *p*=0.03), time spent in medium activity (5.6 ± 2.4, *p*=0.02) and lower percentage of time inactive (78.5 ± 5.9, *p*=0.03). Conversely, there was no difference in percent of time spent in low (12.3 ± 3.7, *p*=0.38) or high activity (1.3 ± 1.5, *p*=0.06).

Adults who did not require ambulatory assistance, such as a cane or walking frame, were more active than those who did as measured by steps per day (4588 ± 1762.4, *p*=0.0003), minutes active (294 ± 81, *p*=0.004), percent of time in medium activity (5.5 ± 2.2, *p*=0.0002) and high-level activity (1.2 ± 1.3, *p*=02). They also exhibited lower percentages of time inactive than those needing assistance (79 ± 5.6, *p*=0.003). Adults who did not require support services from their assisted living home completed a higher number of steps per day (4807.1 ± 2013, *p*=0.02) and spent a greater portion of time doing medium-intensity activity (5.8 ± 2.6, *p*=0.02) than those who required services such as home cleaning (see Table 3).

Multiple regression analyses revealed an inverse relationship between age and activity levels: Younger residents had higher step counts per day (*p* ≤ 0.001), minutes active (*p*=0.001) and a higher percentage of time spent in medium activity (*p* ≤ 0.001). As age decreased, the percentage of time spent in high activity increased (*p*=0.01), while minutes inactive increased with older age (*p* ≤ 0.001). Residents with lower BMI values exhibited more steps per day (*p* ≤ 0.001), more minutes active (*p* ≤ 0.001), in high (*p*=0.02), low (*p*=0.01) and medium activity (*p* ≤ 0.001). High BMI was associated with higher percentages of minutes inactive (*p* ≤ 0.001). There were no relationships evident between activity and level of education or cognitive function. A direct relationship was observed between quality of life and the percentage of time spent in high-intensity activity (*p*=0.04), but not for any other activity measure (see Table 4).

### 3.3. Residents' Perceptions of Living a Physically Active Life

**Figure 1:**
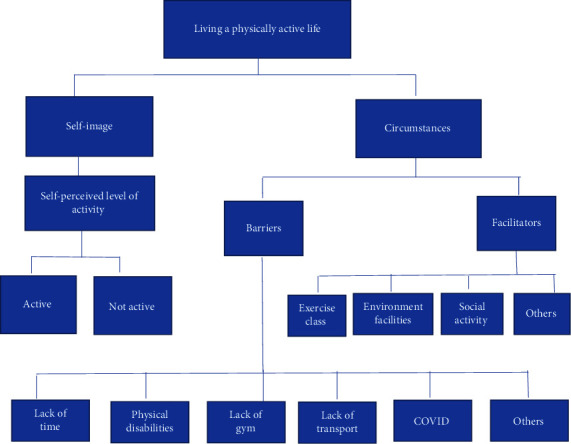
Resident perceptions of influences on living a physically active life.

The major theme to emerge from the interviews centred on the exploration of ‘Living a physically active life.' During this analysis, two categories of primary overarching perspectives were found: (1) self-image and (2) circumstances (see Figure 1)

### 3.4. Self-Image as an Active Elder

Residents' comments reflected the influence of their self-image on their perceptions of personal PA. Self-image encompasses individuals' beliefs and perceptions about themselves, including how they view their own identity and the process of maintaining or changing this self-image [36].

#### 3.4.1. Resident Perceptions of Being Active or Non-Active

Residents discussed the various types of PA they engaged in. This included walking (*n* = 32, 91.4%), with 15 residents (43%) incorporating other activities such as gym workouts, Pilates, tennis, golf, swimming and yoga.

##### 3.4.1.1. I'm Active

Twenty-two residents (63%) reported that they thought they were active and believed that they did enough exercise like walking every day, going gym, swimming and dancing [R34] who confidently stated ‘I walk every morning, walk 2 km every morning and I do yoga on a Thursday morning. And that's about all I do. But I go out a lot.' As well as [R33] said (Yes, I am very active).

##### 3.4.1.2. I'm Active for My Age

Some residents considered their level of activity in the context of their age. Five residents (14.2%) believed they were sufficiently active and believed they did their best considering their age. This sentiment was exemplified by [R15] who confidently stated, ‘I would say sure (I'm active) for my age,' and [R4] who remarked, ‘Yes, I do (believe I am active for my age).' Others expressed that increasing age is a normal part of life and accepted this as a reasonable justification for their inactivity. [R11] said ‘I have got to accept the fact that I am getting older.'

##### 3.4.1.3. I Could Be More Active

Two residents (6%) did not describe themselves to be either active or inactive but believed that they could be more active [R23] enhanced this statement (I could be more active).

##### 3.4.1.4. I'm Not Active

Overall, 6 residents (17.1%) described being inactive because their level of activity was low being limited to such as backward and forward home walking, shopping, walking to café and exercise in bed, for instance, [R26] when questioned about her level of activity she, said (Not typically). However, one resident who thought she was inactive walked 40 min, 3 times a week and participated in dancing once a week.

### 3.5. Physical Activity Is Related to Health

Although all residents expressed recognition of the importance of PA to maintain their health and keep them independent, 6 (17.1%) believed they were not active and attributed this to certain health conditions they were dealing with. For example: [R1] said ‘But [I] also don't feel I'm physically well enough to do what I used to do.' Some felt that it would be necessary to become a younger, healthier self in order to be active again. For instance, [R17] stated that ‘[I'm] Not [active] now. I used to be, but not now because (of) my knees.' And one of the residents [R3] expressed the desire to improve their physical condition: ‘My feet will be better, have better feet.'

### 3.6. Circumstantial Influences on Physical Activity

All residents had been living in their assisted living homes for enduring periods of time and were asked about the positive and negative factors impacting their level of activity. Particularly, questions were asked to explore the facilities and amenities provided by the assisted living home that promoted and encouraged PA. Likewise, questions were asked to identify any obstacles or challenges faced that prevented residents to remain active within their assisted living homes.

#### 3.6.1. Circumstances That Encouraged Physical Activities

Fifty-seven percent of residents expressed the belief that facilities available within their assisted living homes played a significant role in keeping them active and contributed to their overall well-being. Even if not utilised now, they had intentions to potentially benefit from them in the future.

##### 3.6.1.1. Onsite Facilities

One resident specifically mentioned that a positive feature was the exercise classes offered. [R32] remarked:

‘They have a good exercise class here, but as I am a full-time member of a gym, I've been a member of a gym for 30 years, and so I attend at least three, usually four times a week with that. I don't need the classes here. But when I stop being a member of the gym, which I might do [when] I get old, I'll have the classes here.'

While [R20] mentioned: ‘There are some activities on the top floor if you wish to participate.'

##### 3.6.1.2. Location

Furthermore, some residents emphasised the importance of the location of the facility in terms of enhancing exercise opportunities [R21] stated:

‘Probably yes, where we lived before, I probably did less walking because you would get the car to go somewhere. Whereas here, I feel I am walking more even to catch light rail. We just walk across (the road) to the downtown and do whatever want to do.'

And [R25] acknowledged the pleasantness of the location by saying:

‘Yeah … closeness to the walking tracks … You're close to the beach, you can jump on the tram, go down to either [of] the beaches and get lovely walks. You can also go in the water if you want to go down (to) the beach. Yeah, no, I think it's (a) pretty good spot that way.'

##### 3.6.1.3. Socialising

Residents talked about how social activities provided opportunity for physical activity, and this could be facilitated by facility organised activities, friendship with other residents, or with family members.

##### 3.6.1.4. Fellow Residents

Additionally, incidental activity was encouraged through social gatherings. [R34] expressed her love for social activities:

‘It's a great advantage, it's lovely here. I didn't want to live in an apartment, because I always said that you close the door, and you could be dead in bed for three days and nobody know. Whereas this setup, they call it a village, and they encourage people to interact socially. And everybody's very supportive of one another. We wear name tags, and we've got social activities upstairs…. So, it's an excellent setup. And that's the only reason why I came here.'

And [R6] noted: ‘Things I enjoy. Talking to (the) lady over the road.'

##### 3.6.1.5. Family

However, some residents believed that the social support from family rather than the facility was instrumental in promoting activity. When asked what other things make you more active, [R9] mentioned: ‘No, I cannot think I can't think of any… (but) my wife encourages me.'

#### 3.6.2. Circumstances That Discouraged Physical Activity

##### 3.6.2.1. Lack of Onsite Facilities

Conversely, lack of facilities was perceived as a limitation to PA. One frequently mentioned drawback was the lack of a gym in three of the facilities. [R1] expressed, ‘There isn't any (gym) here … (and there) aren't any exercise groups here in the village.' While [R11] mentioned, ‘If they had a gym, yes (I would use it).'

##### 3.6.2.2. Transport

Transportation issues were identified as another barrier. [R7] stated that:

‘I would still drive, yeah, but limited, (I'm) on limited driving. If anybody invited me to do something out of my comfort zone (for) driving, I do not. … look if I could travel farther, I suppose I'd find more classes.'

And [R8] further confirmed this as an issue by mentioning the absence of transport:

‘I don't have transport, I (rely) on the buses and the trains, and … well, yes, if I had the transport, I was going to go, see about going to yoga.'

##### 3.6.2.3. Other Priorities

Some residents mentioned competing priorities for time as an obstacle to being active. They believed they were busy and had limited time for PA because they had other demands. [R13] mentioned, ‘If I could find some more time (I'd be more active),' while [R15] stated that he spent most of his time visiting his friend:

‘But I think I am motivated enough. And as I say, well, I look after my friend over the hill. At the moment now I am spending three nights in my own unit and four nights in hers. And I do a bit of cooking for her.'

##### 3.6.2.4. Physical Disabilities

Physical disabilities were also cited as a hindrance to being active. [R10] mentioned ‘Oh, I've got a bad back.' [R12] said:

‘I thought I would be happy I could have a hip replacement, similar to what I had when I broke the hip on the other side. But my GP thinks it is inactivity. And it is more muscular. That is preventing me from doing things.'

##### 3.6.2.5. The COVID-19 Pandemic

One significant reason affecting residents' lives, as a whole, was the COVID-19 pandemic. [R5] stated:

‘Evidently, they used to have (exercise classes) here, but they haven't because of the COVID and all that, (and) they've never ever got back to it.'

[R12] also attributed her inactivity to the pandemic as well:

‘Well, at the moment, because of COVID there's nothing going on. We do not have anything. But in most places, they would have bus trips, and things like that, but there is nothing here like that at the moment.'

Additionally, residents discussed other barriers related to their personal affect or attitude. For example, [R4] expressed, ‘(I lack) motivation sometimes,' and [R8] mentioned, ‘It is just me.'

### 3.7. The Perceptions of Residents Compared With Measured Activity Levels

Although 83% of residents believed they were active, accelerometer data revealed only 4 undertook 7000 or more steps in regular PA, which is considered a reasonable activity level for maintaining good health in those aged over 65 years [12]. Six residents believed themselves to be inactive; however, measured data on their activity disputes this perception. The five residents with the lowest level of activity all reported that they believed they were active because they do exercise, go to the gym and walk. However, these five residents achieved, on average, less than 2000 steps per day. It is worth noting that these residents were among the oldest in the study, with ages of 90 or more years and therefore their perception was most likely in relation to what they believed to be active for their age.

## 4. Discussion

This is the first study to report both objective measures of activity and qualitative reports of activity in a population of vulnerable older adults who live in assisted living residences in Australia. The research revealed that among older adults, some felt confident in their level of physical activity, while others viewed themselves as inactive, often attributing this perception to their age. Nonetheless, it was evident that these perceptions of activity frequently did not correspond with the current activity guidelines and is perhaps indicative of possible social norms surrounding aged physical activity levels. Residents reported numerous barriers that prevented them from being active. This ranged from individual restrictions such as lack of time, COVID-19 precautions and physical disabilities, to factors associated with the facility itself, such as the lack of an onsite gym and transport, physical environment, lack of supported social activities and exercise classes. Additionally, we discovered that the geographical location, gender, age, not using mobility aids or support services, and comorbidities can impact on older adults' activity. However, marital status and quality of life were not associated with activity levels.

As anticipated, older adults who were younger in age tended to be more physically active compared to others. This aligns with previous research that has shown a decrease in daily activity as age increases [37, 38]. There is less consistency in the literature regarding other variables in older adults and our subpopulation of those living in assisted living housing. Our findings confirm earlier studies highlighting that a higher BMI is associated with lower levels of activity [39], as is the presence of comorbidities and chronic illnesses [40].

Conversely, in our cohort, we observed that females exhibited a higher level of activity than males while other research suggests that women are generally less active due to various psychological factors [41]. Also, we did not find any significant difference in the activity levels of lone livers compared to those living with a spouse. However, another study reported that marital status can have an effect on residential satisfaction, which in turn may improve quality of life and subsequently enhance activity levels [42].

Our study did not find any significant effect of education on PA, although a separate study in the community has shown that lower levels of education among those over 65 years old may impact their ability to understand and act upon health-related information, including the benefits of engaging in PA [40]. It is difficult to explain this variability in findings although we speculate that our populations differ in that they are potentially more vulnerable in terms of frailty and overall, less active. A previous study has reported that residents who live in housing like assisted living homes are less active than those who live in their own homes or apartments [40], the physical activities associated with maintaining one's own home, such as housework and lawn care, contribute to higher activity levels in older adults [40].

We identified that older adults living in assisted living homes who engage in high-intensity activities reported higher quality of life and overall satisfaction, consistent with the studies conducted in institution houses with independent older adults [43]. Caution must be taken not to assume that a higher level of activity is causative of this finding as the converse may be true. Our findings indicate that individuals residing in facility 4 exhibited higher levels of PA compared to older adults in the other 3 facilities. It is worth noting that these individuals were the youngest participants in our study. This could be attributed to the relatively recent building of facility 4, enabling its residents to have moved in more recently. Conversely, the residents in other facilities had been residing in these residences for 2 decades or even longer.

The qualitative findings highlight that many older adults emphasise the importance of living in a location that is close to necessary services, enabling them to be active and independent without having to consider transportation or distance. This finding aligns with a previous study that explored how location can support the physical, mental and social well-being of older adults, ultimately promoting greater activity levels [44]. Additionally, residents in assisted living homes expressed the desire for access to exercise classes, recognising the positive impact such activities can have on their overall activity levels. This supports previous research that suggested providing free exercise classes in assisted living facilities can contribute to the physical health of older adults [45]. The current study also revealed the importance of social activities in enhancing the happiness of older adults living in assisted living homes. Social interaction and enjoyment were the purpose of some residents attending exercise classes, given that loneliness is one of the negatively reported aspects of living in residential/supported care, the importance of offering this service is further highlighted. However, due to the COVID-19 pandemic, some of these activities had been discontinued, which was not welcomed by many residents. Research has shown that loneliness is one of the negatively reported aspects of living [46]. Finally, some residents mentioned that they were not gym enthusiasts and preferred activities like walking to stay active, yet no reported organised walking groups within the facilities were reported.

This study has a number of notable strengths. Firstly, this is the first study utilising a socioecological model to explore the level of activity as well as perception of activity in a group of older adults living in assisted living homes. The concurrent triangulation of evidence has facilitated a more comprehensive analysis and enabled the verification of perceived activity levels through objective measures of physical activity. Secondly, pursuing broad sampling led to the inclusion of a facility with a different design and type of location allowing us to make a connection as to how geographic location of assisted living homes may influence PA rather than just personal intrinsic characteristics and attitudes. Whilst our study provides valuable information for this population group, it is important to acknowledge that this study has some weaknesses. It was conducted specifically in four assisted living homes in Australia; consequently, the generalisability of the results to other assisted living home populations, in other settings, regions and countries, may be limited. With this baseline, future research could investigate the implementation of different social and physical activity interventions and measure the effects longitudinally to assess health-related impacts.

In conclusion, our study provides valuable insights into the factors influencing the activity levels of older Australian adults in assisted living residences. These findings have the potential to enhance the independence of older individuals and offer suggestions to assisted living facilities on how to provide appropriate amenities to promote residents engagement in PA in order to extend independence and minimise health risks associated with inactivity.

## Figures and Tables

**Table 1:** Characteristics of the residents.

**Characteristic**	**Range**	**Mean ± SD**

Age (years)		
Total	71–99	83.8 ± 7.20
BMI (weight/height^2^)		
Female	16.78–35.92	26.29 ± 4.62
Male	19.23–35.97	26.29 ± 4.68
Total	16.78–35.97	26.26 ± 4.66
Cognitive function		
MMSE score	23–30	28.84 ± 1.59
QoL		
OPQOL-35 score	99–160	141.2 ± 12.88
Comorbidity		
CCI score	0–8	2.31 ± 1.68

**Gender**	***N***	**Percentage (%)**

Female	24	68.5
Male	11	31.4
Marital status		
Married	14	40
Widow	14	40
Widower∗	3	8.5
Single	4	11.4
Education		
Did not complete high school	8	22.8
High school certificate	5	14.2
TAFE	12	34.2
Bachelor's degree	7	20.0
Postgraduate degree	3	8.5
Ambulatory assistance		
No	25	71.4
Yes	10	28.5
Services		
Home cleaning	20	57.1
Podiatry	2	5.7
Physiotherapy	4	11.4
No need for services	13	37.14

Abbreviations: BMI, body mass index; CCI, Charlson comorbidity index; MMSE, mini-mental state examination; N, number; OPQOL-35, older people's quality of life questionnaire.

^∗^Two widowers are partners.

**Table 2:** Activity averaged for all residents across 5 days.

Step per day (mean ± SD)	Minutes active	Percent inactive (%)	Percent time in low active (%)	Percent time in medium active (%)	Percent time in high active (%)	Cadence average (mean ± SD)	Max 60 (mean ± SD)
3940.31 ± 1849.65	271.82 ± 80.66	81	12	6	1	14.06 ± 3.54	18.30 ± 9.88

*Note:* Cadence average: average cadence based on all minutes with at least 1 step. Max 60: average cadence of the most intensive continuous 60 min of the day.

**Table 3:** Activity levels according to demographic variables.

Variables	*N*	Steps per day Mean ± SD	Minutes active Mean ± SD	% No activity	% Low activity	% Medium activity	% High activity
Total sample	35	3940.31 ± 2134.7	271.82 ± 100.7	81 ± 6.9	12 ± 4.5	6 ± 2.8	1 ± 1.4

*Gender*							
Female	24	4096 ± 1634	287 ± 68.3	79.6 ± 4.8	12.7 ± 2.8	4.8 ± 2.2	1/1.2
Male	11	3556 ± 2296.2	235 ± 97	83.1 ± 6.7	9.8 ± 3.6	4.2 ± 2.8	0.81 ± 1.4
*p* value		0.43	0.07	0.08	0.01∗	0.53	0.70

*Comorbidities*							
None	4	4193.2 ± 558.1	270.3 ± 33.2	80.5 ± 23.8	11.2 ± 0.95	5.2 ± 1.5	1.2 ± 0.5
One disease or more	31	3892 ± 1958.4	270.3 ± 85.2	80.7 ± 5.9	11.9 ± 3.5	4.5 ± 2.5	0.90 ± 1.3
*p* value		0.76	0.99	0.92	0.72	0.61	0.62

*Marital status*							
Not alone	16	3879.9	272.6	80.5	12.1	4.6	0.87
		1986.8	81.8	5.7	3.5	2.3	1.3
Alone	19	3964.8	268.4	80.8	11.5	4.6	1
		1780.3	81.9	5.7	3.2	2.5	1.2
*p* value		0.89	0.88	0.86	0.64	0.94	0.77

*Facility*							
Villa (1–3)	3	3179 ± 1488	245 ± 69.1	82.5 ± 4.8	11.3 ± 3	3.8 ± 2.1	0.57 ± 0.9
Apartment (4)	1	4814 ± 1884.1	301 ± 84.7	78.5 ± 5.9	12.3 ± 3.7	5.6 ± 2.4	1.3 ± 1.5
*p* value		0.007	0.03∗	0.03∗	0.38	0.02∗	0.06

*Ambulatory assistance*							
No aid	25	4588 ± 1762.4	294 ± 81	79 ± 5.6	12.2 ± 3.4	5.5 ± 2.2	1.2 ± 1.3
Aid required	10	2271.4 ± 619	212 ± 42	85 ± 2.9	10.8 ± 3	2.4 ± 0.8	0.2 ± 0.4
*p* value		0.0003∗	0.004∗	0.003∗	0.26	0.0002∗	0.02∗

*Services*							
No services	13	4807.1 ± 2013	295.4 ± 99.1	78.9 ± 6.8	12 ± 4	5.8 ± 2.6	1.4 ± 1.5
Services received	22	3405.3 ± 1568.2	255.4 ± 66	81.8 ± 4.6	11.7 ± 2.9	3.9 ± 2	0.63 ± 1
*p* value		0.02∗	0.15	0.14	0.82	0.02∗	0.06

^∗^ *p* < 0.05 (Significant difference).

**Table 4:** Multiple regression *p* values for activity measures and demographic variables.

Variables	Step per day	Minutes active	Percent inactive	Percent low activity	Percent medium activity	Percent high activity
Age	0.001∗	0.001∗	0.001∗	0.25	0.001∗	0.01∗
Education	0.72	0.31	0.38	0.35	0.78	0.94
BMI	0.001∗	0.001∗	0.001∗	0.01∗	0.001∗	0.02∗
MMSE	0.88	0.35	0.25	0.38	0.66	0.78
QOL	0.20	0.13	0.12	0.20	0.72	0.04∗
Comorbidity	0.06	0.01∗	0.01∗	0.01∗	0.12	0.13

^∗^ *p* < 0.05 (Significant association).

## Data Availability

The deidentified data that support the findings of this study are available from the corresponding author upon reasonable request.
